# Exosomal hsa-miR199a-3p Promotes Proliferation and Migration in Neuroblastoma

**DOI:** 10.3389/fonc.2019.00459

**Published:** 2019-06-12

**Authors:** Jing Ma, Min Xu, Minzhi Yin, Jie Hong, Haoyan Chen, Yijin Gao, Chenjie Xie, Nan Shen, Song Gu, Xi Mo

**Affiliations:** ^1^Department of Pathology, Shanghai Children's Medical Center, Shanghai Jiao Tong University School of Medicine, Shanghai, China; ^2^Department of General Surgery/Surgical Oncology Center, Shanghai Children's Medical Center, Shanghai Jiao Tong University School of Medicine, Shanghai, China; ^3^State Key Laboratory for Oncogenes and Related Genes, Key Laboratory of Gastroenterology and Hepatology, Ministry of Health, Division of Gastroenterology and Hepatology, Renji Hospital, Shanghai Cancer Institute, Shanghai Institute of Digestive Disease, Shanghai Jiao Tong University School of Medicine, Shanghai, China; ^4^Department of Hematology and Oncology, Shanghai Children's Medical Center, Shanghai Jiao Tong University School of Medicine, Shanghai, China; ^5^Shanghai Children's Medical Center, Pediatric Translational Medicine Institute, Shanghai Jiao Tong University School of Medicine, Shanghai, China; ^6^Department of Infectious Diseases, Shanghai Children's Medical Center, Shanghai Jiaotong University School of Medicine, Shanghai, China; ^7^Department of Rehabilitation, Xinhua Hospital, Shanghai Jiao Tong University School of Medicine, Shanghai, China

**Keywords:** neuroblastoma, exosome, microRNA, hsa-miR199a-3p, *NEDD4*, proliferation, migration, biomarker

## Abstract

Neuroblastoma (NB) is the most common pediatric extra-cranial solid tumor with heterogeneous characteristics, and the prognosis of patients with high-risk NB is usually poor. Discovery of novel biomarkers for early detection and investigation of the underlying mechanisms governing invasion and metastasis of NB are urgently needed. Recently, exosomal microRNAs (miRNAs) have been shown to play vital regulatory or communication roles in the process of various types of cancers. However, the roles and mechanisms of exosomal miRNAs in NB remain unknown. Thus, the present study aims to investigate the detailed functions of tumor-derived exosomal miRNAs in progression and migration of NB *in vivo* and *in vitro*. By examining different exosomal miRNA expression profiles in the plasma of NB patients, we identified that the expression of hsa-miR199a-3p from exosomes was significantly upregulated, which was correlated with the severity of NB patients. Furthermore, we confirmed that exosomal hsa-miR199a-3p could facilitate proliferation and migration of NB via regulating *NEDD4* expression. In summary, our data, for the first time, revealed that exosomal hsa-miR199a-3p could promote tumor proliferation and migration via decreasing *NEDD4* expression in NB, suggesting that exosomal hsa-miR199a-3p may be applicated as a fast, easy, and non-invasive detection biomarker and contribute to the development of novel therapeutic strategies for NB in the future.

## Introduction

Neuroblastic tumors (NTs), including neuroblastoma (NB), ganglioneuroblastoma, intermixed (GNBi), ganglioneuroblastoma, nodular (GNBn), and ganglioneuroma (GN), are the most common pediatric extra-cranial solid tumors (1). They arise from the embryonic sympathoadrenal neural crest and range from immature to differentiated mature tumors. Based on the International Neuroblastoma Pathology Classification (INPC), GNBi, and GN are relatively differentiated mature variants of NT, while NB represents the immature cancer type (2). As the third most frequent childhood cancer, NB accounts for 7% of the total cases of cancer diagnosed among children aged birth to 14 years old (3). More remarkably, NB is the primary cause of death from pediatric cancers in children between 1 and 5 years old and leads to approximately 13% of pediatric cancer mortality (4). However, the survival rates of NB at different risk categories are markedly variable. The 5-year survival rates of NB patients with low- to intermediate-risk are over 90%, compared to only 40–50% in patients with high-risk NB (5). Because of rapid invasion and metastasis, the prognosis of NB patients with high-risk NB is poor even using some aggressive therapeutic strategies. Easy and fast detection in NB patients can contribute to their diagnosis and monitoring responses to therapy. Therefore, discovery of novel diagnostic and prognostic biomarkers in NB and comprehensive investigation of the mechanisms governing its invasion and metastasis are urgently needed.

Recently, there have been renewed interests in the extracellular vehicles (EVs) because of their numerous functions in physiology and pathology. Exosomes, small EVs (40–100 nm in diameter) formed inside the endosomal compartments, contain various RNAs such as mRNAs and miRNAs. Although the biological functions of exosomal miRNAs are not yet completely clear, they have been reported to play vital roles in the regulation of tumorigenesis and tumor development (6–8). A recent study has demonstrated that *MYCN*-amplified NB cells can secrete some exosomal miRNAs which may play potential roles in cancer progression via the change of tumor environment (9). Moreover, circulating exosomal miRNA-21 and miRNA-155 have been reported to be involved in NB resistance to chemotherapy through a novel exosomal miR-21/TLR8-NF-κB/exosomal miR-155/TERF1 signaling pathway (10). However, few studies have detected the specific circulating exosomal miRNA profiles in the peripheral blood samples from patients with high-risk NB and investigated the mechanisms of specific exosomal miRNAs in the NB invasion and metastasis.

In the present study, an extensive investigation on the circulating exosomal miRNA profiles of NB patients paired with GNBi and healthy individuals was performed using miRNA-sequencing. The purpose of this study is to identify suitable circulating exosomal miRNAs as novel biomarkers that can improve early diagnosis in NB, and to investigate the underlying molecular mechanisms involved in these exosomal miRNAs.

## Materials and Methods

### Patients and Clinical Samples

A total of 17 NB patients, 6 GNBi patients as well as 7 age- and sex-matched healthy individuals were recruited in Shanghai Children's Medical Center (SCMC) from January 2017 to September 2017. According to the INPC, all enrolled patients were newly diagnosed as NB and GNBi with histological provement, and had not received chemotherapy or radiotherapy. The clinic-pathological characteristics of all the patients were listed in the Tables S1, S2.

Two milliliter of peripheral blood was collected in EDTA anticoagulant tubes. Plasma was obtained after a 20-min centrifugation at 1,500 × *g* and a second 10-min centrifugation at 2,000 × *g* to remove the blood cells. The plasma samples were apportioned in aliquots and stored at −80°C until further use.

The study was approved by the Institutional Review Board and the Ethics Committee of SCMC (SCMCIRB-K2017042), and written informed consent was obtained from the parents of each participant.

### Cell Culture

Three human NB cell lines [i.e., SK-N-SH, SH-SY5Y, SK-N-BE(2)] and human umbilical vein endothelial cell line (HUVEC), human embryonic kidney cell line (HEK293), and human fibroblast cell line (MRC5) were used in this study. All cell lines were obtained from Cell Bank of Chinese Academy of Sciences (Shanghai, China) except for MRC5, which was kindly provided by Dr. Jing Ye (Ruijin Hospital, Shanghai Jiao Tong University School of Medicine). The cells were cultured in exosome-free conditioned medium containing Dulbecco's modified Eagle's medium (DMEM) or Dulbecco's modified Eagle's Medium: Nutrient Mixture F-12 (DMEM/F12) supplemented with 10% fetal bovine serum (FBS) and incubated at 37°C in a humidified incubator with 5% CO_2_. The FBS was depleted of bovine exosomes by overnight ultracentrifugation at 120,000 × *g* followed by filtration with 0.22-μm filter.

### Isolation of Exosomes From Plasma Samples

Plasma exosomes were isolated and purified using the exoRNeasy serum/plasma midi kit (Qiagen, USA) according to the manufacturer's protocol. Briefly, 1 volume of buffer XBP was mixed with the pre-filtered plasma sample and added onto the exoEasy spin column. After centrifugation, the pelleted fraction was re-suspended in nuclease-free water for later analysis or subjected to RNA extraction immediately. All centrifugation steps were performed at 4°C.

### Isolation of Exosomes From Cell Culture Supernatants

Exosomes from cell culture supernatants were isolated as described previously (11). Briefly, conditioned medium was harvested 4 days after cell seeding. After centrifugation at 2,000 × *g* and 10,000 × *g* to remove the cells and cellular debris, respectively, the supernatant was centrifuged at 100,000 × g for 70 min to collect the exosome fraction. The crude exosome pellets were washed in phosphate buffer saline (PBS), centrifuged at 100,000 × *g* for 1 h, and was then re-suspended in PBS. All centrifugation steps were performed at 4°C.

### Characterization of Isolated Exosomes

To confirm the purification of the isolated exosomes, transmission electron microscopy (TEM), Nanosight analysis, flow cytometry analysis, and Western blotting were performed. In Brief, 10 μL of isolated exosomes was mounted on copper grids and fixed with 1% glutaraldehyde. The grids were stained with 3% (v/v) phosphotungstic acid in ddH_2_O and the samples were examined immediately with JEM1230 TEM (JEOL, Japan) at a voltage of 80 kV. Moreover, the size distribution of the exosomes was evaluated with Zeta sizer Nano series-Nano-ZS (Malvern Instruments, UK) using Nanoparticle Tracking Analysis (NTA) software v2.3 (NanoSight Ltd, UK). Furthermore, flow cytometry analysis with an Accuri C6 flow cytometer (BD Instruments, USA) was performed to detect exosomal specific surface antigens (i.e., CD63 and CD81). At last, exosome-specific markers CD63 and calnexin were identified with Western blotting. Briefly, 10 μg of the exosomal proteins were separated on 10% sodium dodecyl sulfate–polyacrylamide gel electrophoresis (SDS-PAGE) gels and transferred to polyvinylidene difluoride (PVDF) membrane (GE Healthcare, USA). Blocked with 5% non-fat milk in PBST (PBS containing 0.1% TWEEN-20), the membrane was incubated with the primary antibodies against CD63 (Abcam, ab68418) and calnexin (NOVUS, NB100-1974), respectively, at 4°C overnight. The membrane was then washed 3 times with PBST and exposed to horseradish peroxidase (HRP)-conjugated donkey anti-mouse IgG (1:1000 dilution, Santa Cruz Biotechnology) for 1 h at room temperature. After 3-time washes with PBST, antibody binding was detected by enhanced chemiluminescence substrates (Millipore, USA) and visualized with ImageQuant LAS 4000 mini densitometer (GE Healthcare Life Sciences, USA).

### Extraction of RNA From Exosomes

The exosomal RNAs were extracted using Qiazol and RNeasy MinElute Spin Column (Qiagen, USA) following the manufacturer's user guide. RNA concentration was determined using Nanodrop (Thermo Fisher Scientific, USA). The content and quality of exosomal RNAs were determined using the 2100 Bioanalyzer instrument (Agilent technologies, USA).

### miRNA Library Construction and Sequencing Data Analysis

Deep sequencing was performed on all exosomal RNAs from the plasma samples. 18–30 nt miRNAs were first size-selected with PAGE gel and reverse transcribed to cDNA through adaptor-specific primers. PCR amplification was then carried out and eluted in nuclease-free H_2_O. The quality and yield of miRNA libraries were assessed using the Agilent 2100 Bioanalyzer instrument. The PCR products of 100–200 bp were then separated from primer-dimers and other products with PAGE gel.

The expression levels of miRNAs in the constructed libraries were quantified using the BGIseq-500 platform (BGI, China). The raw data cleaning analysis included removing the low-quality tags and 5' adaptor contaminants from the 50-nt tags. Based on miRbase and Rfam databases, the clean tags were annotated into different categories to predict the novel miRNAs and seed edit of potential known miRNAs. Prediction for target miRNAs and the target genes accordingly were finally performed using Gene Ontology (GO) enrichment and Kyoto Encyclopedia of Genes and Genomes (KEGG) pathway analysis.

### Verification of miRNA Expression With Quantitative Real Time PCR (qRT-PCR)

Qiagen miScript SYBR Green PCR assay was used for miRNA quantification in accordance with the manufacturer's instructions. In brief, the reverse transcription of miRNAs from the plasma samples, cell culture supernatants and tissues were performed with miScript II RT kit. The specific primers of miRNAs were designed by Qiagen, and exogenous cel-miR-39-3p was used as control. qRT-PCR was performed in triplicate using the CFX Connect™ Real-Time System (Bio-Rad, USA) under the following conditions: 95°C for 15 min, 40 amplification cycles of 94°C for 15 s, 55°C for 30 s, and 70°C for 30 s. The data are presented as 2^−Δ*ΔCT*^. In the analysis of exosomal miRNAs, the first sample collected in the healthy controls was used as 1 for normalization. In the analysis of miRNAs of the tumor tissues, the first sample collected in the low-risk patients was used as 1 for normalization.

### Cell Transfection

The specific mimics for hsa-miR199a-3p and negative control were designed and synthetized by Ribobio (Ribo, China). The sequence of hsa-miR199a-3p mimic was sense 5′-ACAGUAGUCUGCACAUUGGUUA-3′ and antisense 5′-UAACCAAUGUGCAGACUACUGU-3′. Sequence of miRNA mimic used as control miRNA was: sense 5′-UUUGUACUACACAAAAGUACUG-3′, antisense 5′-CAGUACUUUUGUGUAGUACAAA-3′. The hsa-miR199a-3p mimic and negative control were transfected into SK-N-SH cells using riboFECT™ CP transfection reagent (Ribo, China) following the manufacturer's instructions. In addition, *NEDD4* cDNA with HA tag at the C-terminus was synthesized and constructed into pcDNA3.1 vector (Generay Biotech Co., China). Both pcDNA3.1-*NEDD4* and empty pcDNA3.1 plasmids were transfected into cells with Lipo3000 transfection reagent (Thermo Fisher Scientific, USA) following the manufacturer's instructions.

### BrdU Assay

The effects of the exosomal miRNAs on cell proliferation were also detected using the bromodeoxyuridine (BrdU) cell proliferation assay kit (Roche, USA) following the manufacturer's instructions. Briefly, the cells were firstly incubated with 10 μM BrdU labeling solution for 2 h, and were then fixed and incubated with anti-BrdU-POD for 2 h after removal of the BrdU labeling solution. The absorbance of each well was measured with a Multilabel Counter and normalized to that of the cells at 0 h.

### MTS Assay

The effects of the exosomal miRNAs on cell proliferation were also evaluated with 3-(4,5-dimethylthiazol-2yl)-2,5-dipheiltertrazolium bromide (MTS) Cell Proliferation Assay (Promega, USA) following the manufacturer's instructions. In brief, 0, 24, 48, and 72 h after miRNA transfection, 20 μL of MTS was added to each well, and the plate was detected by a Multilabel Counter (Bio-Rad Laboratories, USA) after 3 h of incubation. Absorbance at 490 nm of each well was measured and normalized to that of the cells at 0 h.

### Cell Migration Assay

The effects of the exosomal miRNAs on cell migration were assessed using Transwell migration chamber with 8.0 μm pore size polycarbonate membrane (Corning, USA). In brief, 50,000 cells were seeded in the Transwell upper chamber with 200 μL Opti-MEM medium (Gibco, USA). The lower chamber was filled with 200 μL culture medium supplemented with 20% FBS. After 24, 48, and 72 h, the filters were washed, fixed and stained with Crystal Violet (Sigma-Aldrich, USA). The number of migrated cells onto the lower surface of the filter was counted in five different fields at a 400 × magnification.

### Luciferase Assay

Following transfections of the reporter plasmid, dual luciferase reporter assays (Promega, USA) were carried out according to the manufacturer's instructions. Luciferase activity was measured using a Multilabel Counter (Bio-Rad Laboratories, USA).

### Statistical Analysis

All statistical analysis was performed with IBM SPSS 24.0 statistical software (SPSS Inc., USA) and graphs were generated using GraphPad PRISM 6.0 (GraphPad Software, Inc., USA). The differential expression levels of exosomal miRNAs between different groups were compared using Wilcoxon rank-sum test, and continuous variables were analyzed using the Student's *t*-test. *p* < 0.05 was considered as statistically significant.

## Results

### Exosomal hsa-miR199a-3p Is Highly Expressed in the Plasma of NB Patients

**Figure 1:**
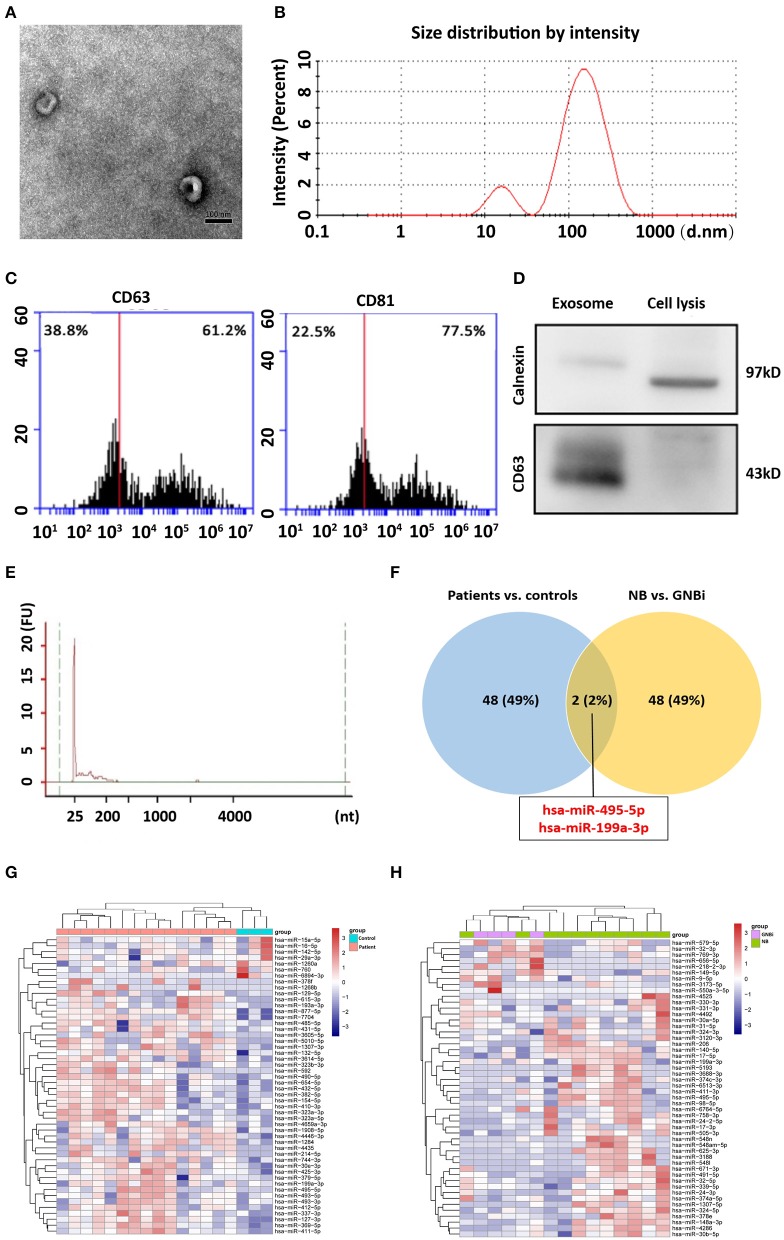
Identification of plasma exosomes and differentially expressed exosomal miRNAs. **(A)** Transmission electron microscopy demonstrated the presence of exosome vesicles with typical cup-shape morphology (size bar 100 nm). **(B)** Analysis of exosomes isolated from plasma by Nanoparticle Tracking Analysis. The average size was 79.42 nm and the peak was 146.4 nm. **(C)** 61.2% particles isolated from the plasma samples showed CD63 positive and 77.5% showed CD81 positive as detected by flow cytometry. **(D)** Confirmation of the exosomes markers with Western blotting indicated the presence of CD63 but the absence of calnexin in exosomes. **(E)** Bioanalzyer trace analysis revealed abundant miRNAs present in the exosomes isolated from the plasma samples. The exosomes presented in **(A–E)** were obtained from NB patient 1. **(F)** A Venn diagram showed that hsa-miR199a-3p and hsa-miR-495-5p overlapped in the top 50 most different miRNAs between NB/GNBi patients vs. healthy individuals and NB vs. GNBi patients (red color indicates up-regulation). **(G,H)** Cluster analysis of exosomal miRNAs among NB, GNBi, and healthy individuals. The top 50 most different miRNAs between NB/GNBi and healthy controls **(G)** as well as those between NB and GNBi patients **(H)** were presented.

Isolated from the plasma of NB patients, the EVs were identified with transmission electron microscope and Nanoparticle Tracking Analysis (Figures 1A,B). In addition, results from flow cytometry analysis demonstrated the existence of exosomal markers CD63 and CD81 (Figure 1C). The presence of the exosomal marker CD63 but the absence of endoplasmic reticulum compartment-specific marker calnexin was also confirmed by Western blotting (Figure 1D). All these results indicated that the isolated EVs were purified exosomes.

**Figure 2:**
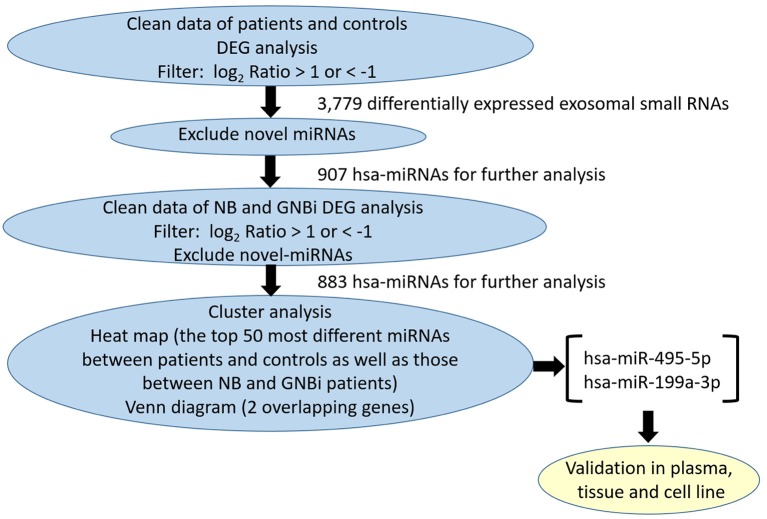
Workflow of the screening procedures used to identify the target exosomal miRNAs.

**Figure 3:**
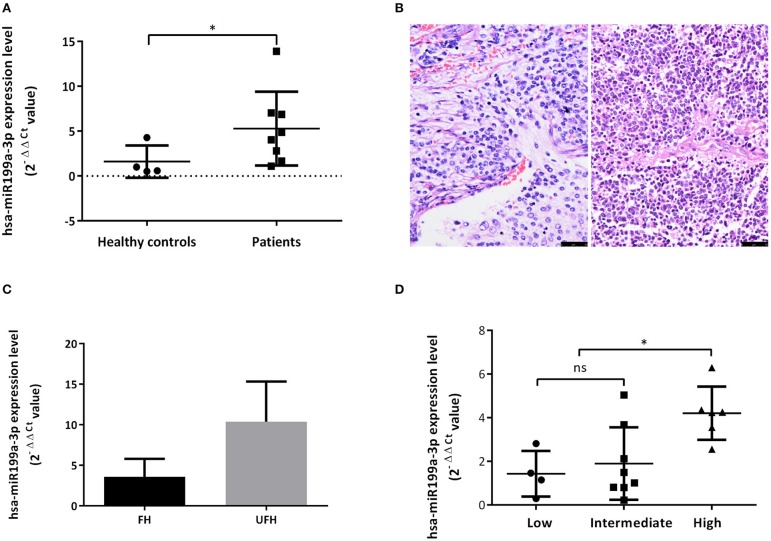
Exosomal hsa-miR199a-3p was highly expressed in the plasma of NB patients and predicted high risk of NB patients. **(A)** The exosomal hsa-miR199a-3p expression levels were significantly higher in the patients with NB/GNBi compared with healthy controls as determined by qRT-PCR. **(B)** Representative images of FH (left) and UFH (right) neuroblastoma tissues were presented after H&E staining (size bar 50 μm). **(C)** Exosomal hsa-miR199a-3p levels in the plasma of the UFH patients (*N* = 2) were 2-fold more than those of the FH patients (*N* = 6). **(D)** hsa-miR199a-3p in the fresh tumor tissues were higher in the COG high-risk group compared with the intermediate- and low-risk group as determined by qRT-PCR. *p < 0.05; ns, not significant.

Bioanalyzer trace analysis revealed abundant miRNAs present in the exosomes isolated from the plasma samples (Figure 1E). Afterwards, next-generation sequencing was performed on these exosomal miRNA samples from NB, GNBi and healthy individuals, respectively. The criteria used to screen differences in miRNA encapsulation in the plasma exosomes between NB/GNBi patients and healthy individuals, as well as those between NB patients and GNBi patients were based on differentially expressed gene (DEG) analysis (log_2_ Ratio > 1 or < −1). A total of 3,779 differentially expressed exosomal small RNAs met these criteria and were selected for further analysis, among which 3,248 small RNAs were up-regulated and 531 small RNAs were down-regulated in the NB/GNBi patients. A Venn diagram showed that only hsa-miR199a-3p and hsa-miR-495-5p overlapped in the top 50 most different miRNAs between NB/GNBi patients *vs*. healthy individuals and NB *vs*. GNBi patients (Figure 1F). Besides, two heat maps of these 50 selected miRNAs demonstrated a gene cluster and sample cluster according to the levels of exosomal miRNAs in the plasma among NB, GNBi and healthy individuals (Figures 1G,H). The screening procedures used to identify the target exosomal miRNAs are shown in Figure 2. qRT-PCR was performed to validate the 2 selected exosomal miRNA candidates in other 8 newly diagnosed NB/GNBi patients and 4 healthy volunteers. We confirmed that only the expression levels of hsa-miR199a-3p, but not the hsa-miR-495-5p were significantly higher in the plasma exosomes from NB patients (*p* = 0.042; Figure 3A), suggesting that plasma exosomal hsa-miR199a-3p may play a vital role in NB progression.

### Upregulated hsa-miR199a-3p Predicts High Risk of NB Patients

To explore whether exosomal hsa-miR199a-3p expression correlates with the severity of NB patients, we divided the patients into groups with favorable histology (FH) and unfavorable histology (UFH) according to the INPC (Figure 3B). The clinico-pathological characteristics of the patients in both groups were comparable (Table S2). Under such classification, the exosomal hsa-miR199a-3p levels in the plasma of patients with UFH were 2-fold higher than those patients with FH (Figure 3C), suggesting that upregulated exosomal hsa-miR199a-3p may serve as an independent risk factor. In addition, the expression of hsa-miR199a-3p in the fresh tumor tissues from 18 patients with NB and GNB were detected. The characteristics of these 18 patients were listed in the Table S3. Even in the fresh tissues, the expression levels of hsa-miR199a-3p were significantly higher in the Children's Oncology Group (COG) high-risk patients than those in the intermediate- and low-risk patients (Figure 3D).

### Exosomal hsa-miR199a-3p Facilitates Proliferation and Migration of NB Cells

**Figure 4:**
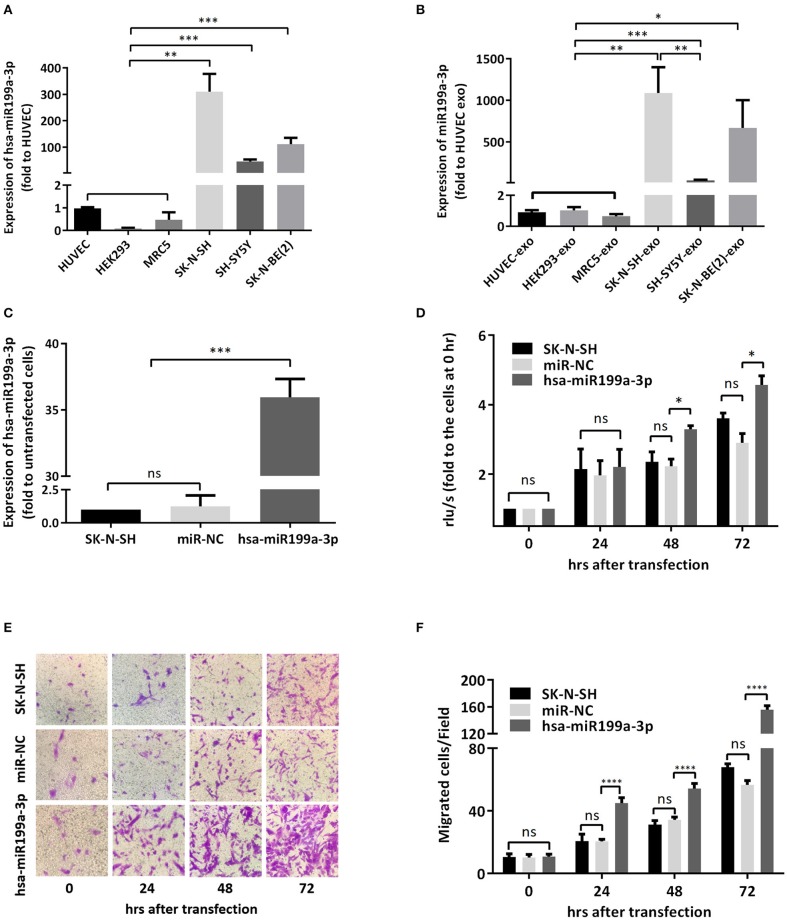
hsa-miR199a-3p facilitated proliferation and migration of NB cells. **(A,B)** The expression levels of hsa-miR199a-3p in the NB cells [SK-N-SH, SH-SY5Y, SK-N-BE(2)] **(A)** and in the exosomes secreted from these cells **(B)** were significantly higher than those in the normal control cells (HUVEC, HEK293, MRC5), as detected by qRT-PCR. **(C)** hsa-miR199a-3p was up-regulated in SK-N-SH cells after transfection with the designed hsa-miR199a-3p mimic. **(D)** BrdU cell proliferation assay demonstrated that over-expression of hsa-miR199a-3p can promote the proliferation of SK-N-SH cell lines 48 and 72 h after transfection. **(E,F)** Transwell assay revealed the up-regulation of exosomal hsa-miR199a-3p can promote the migration of SK-N-SH cells 24, 48, and 72 h after transfection. Representative photographs were shown in **(E)** and quantification of the migrated cells per field was shown in **(F)**. All the data were presented as the mean ± SD from three independent experiments. *p < 0.05; **p < 0.01; ***p < 0.001; ****p < 0.0001; ns, not significant. exo, exosome; NC, negative controls.

To verify whether the significantly higher exosomal hsa-miR199a-3p identified in the plasma of NB patients were due to the high expression of hsa-miR199a-3p in NB tumors, we analyzed the levels of hsa-miR199a-3p in three NB cell lines, including SK-N-SH cells, SH-SY5Y and SK-N-BE(2) and the exosomes secreted from these cells. HUVEC, human fetal lung fibroblast cells (MRC-5), and human embryonic kidney 293 cells (HEK 293) were used as normal controls. As determined by qRT-PCR, the expression levels of hsa-miR199a-3p in both the SK-N-SH cells (309.50 ± 39.17 *vs*. 1.05 ± 0.02, *p* = 0.0014) and the exosomes secreted from SK-N-SH (1,087 ± 178.4 *vs*. 0.90 ± 0.08, *p* = 0.0037) were significantly higher than those in HUVEC cells and their exosomes. Compared to HUVEC, similar phenomena were also observed in SH-SY5Y cells (45.5 ± 4.38, *p* = 0.0005) and SK-N-BE(2) cells (111.5 ± 13.53, *p* = 0.0005), as well as the exosomes [(38.02 ± 3.613, *p* = 0.0005 in SH-SY5Y and 666.1 ± 193.5, *p* = 0.012 in SK-N-BE(2)] secreted from the cells (Figures 4A,B). In contrast, no significant difference of hsa-miR199a-3p has been observed among the three non-NB cell lines, either in the cells or in the exosomes. These results show that compared to the non-NB cell lines, hsa-miR199a-3p were significantly higher expressed in the NB cells and their exosomes, suggesting that the remarkably abundant exosomal hsa-miR199a-3p levels in the plasma of NB patients may be due to its high expression in NB tumors.

**Figure 5:**
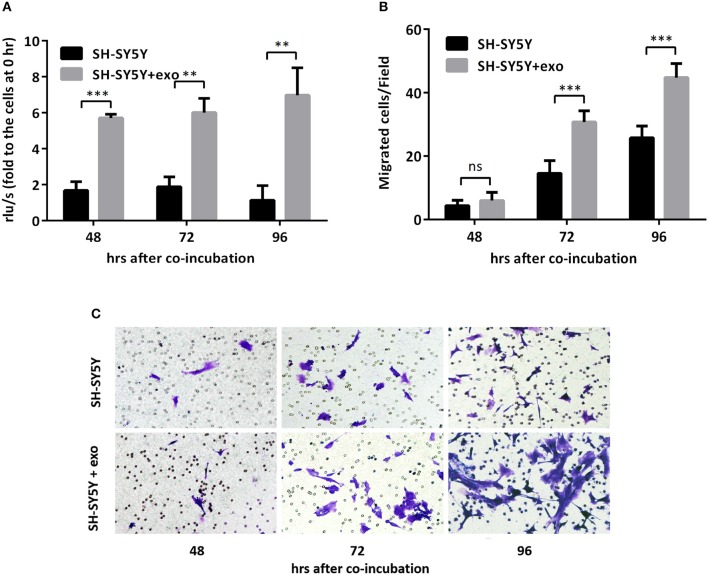
Exosomal hsa-miR199a-3p from SK-N-SH cells facilitated proliferation and migration of NB cells. **(A)** BrdU cell proliferation assay demonstrated that exosomal hsa-miR199a-3p obtained from SK-N-SH cells could promote SH-SY5Y cell proliferation 48, 72, and 96 h after co-incubation. **(B,C)** Transwell assay revealed exosomal hsa-miR199a-3p obtained from SK-N-SH cells could promote SH-SY5Y cell migration 72 and 96 h after co-incubation. Quantification of the migrated cells per field was shown in **(B)** and representative photographs were shown in **(C)**. All the data were presented as the mean ± SD from three independent experiments. **p < 0.01; ***p < 0.001; ns, not significant. exo, exosome.

To further evaluate the functions of exosomal hsa-miR199a-3p in NB progression, hsa-miR199a-3p mimic was synthesized and transfected into SK-N-SH cells. After transfection, the expression level of exosomal hsa-miR199a-3p from SK-N-SH cell culture supernatants was significantly increased (Figure 4C). Moreover, BrdU cell proliferation assay showed that hsa-miR199a-3p overexpression promoted significant proliferation of NB cells at 48 (3.29 ± 0.010 *vs*. 2.23 ± 0.21, *p* = 0.0431) and 72 h (4.57 ± 0.26 *vs*. 2.90 ± 0.27, *p* = 0.0462) after transfection (Figure 4D). Similar results have also been observed when detected by MTS assay (data not shown). In addition, as detected in the Transwell assay, up-regulation of hsa-miR199a-3p significantly increased the migration of NB cells at different time points after transfection (45.00 ± 1.52 migrated cells/field *vs*. 20.40 ± 0.68, 54.20 ± 1.43 *vs*. 34.20 ± 0.86, and 56.60 ± 1.21 vs. 147.40 ± 3.06 at 24, 48, and 72 h respectively, *p* < 0.0001; Figures 4E,F). To further validate the role of exosomal hsa-miR199a-3p in promoting proliferation and migration in NB, exosomes obtained from the culture supernatant of SK-N-SH cells, which contain the highest level of exosomal hsa-miR199a-3p among the three NB cell lines, were used to treat the SH-SY5Y cells, which contain the lowest level of exosomal hsa-miR199a-3p. As expected, hsa-miR199a-3p-enriched exosomes could facilitate the proliferation and migration of SH-SY5Y cells (Figure 5). Taken together, the data indicate that upregulation of exosomal hsa-miR199a-3p significantly increases the proliferation and migration of NB cells.

### hsa-miR199a-3p Inhibits *NEDD4* Expression by Binding to Its mRNA in NB Cells

**Figure 6:**
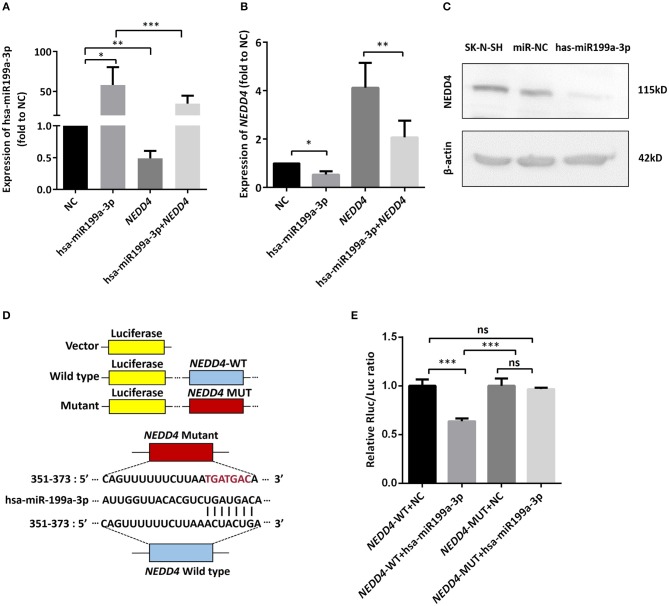
hsa-miR199a-3p inhibited *NEDD4* expression by binding to its mRNA in NB cells. **(A,B)** Expression of hsa-miR199a-3p **(A)** and the mRNA levels of *NEDD4* **(B)** after transfection with hsa-miR199a-3p mimic and *NEDD4* overexpression plasmid separately or in combination, as determined by qRT-PCR. The data showed that the expression of hsa-miR199a-3p was negatively correlated with the *NEDD4* expression levels. **(C)** Western blotting showed the expression of *NEDD4* was down-regulated in SK-N-SH cells after transfection with hsa-miR199a-3p mimic. The figure was a representative from three independent experiments. **(D)** Vectors with or without wild type or mutant 3′-untranslated regions (UTR) of *NEDD4* mRNA and diagram of the binding site of 3′-UTR of *NEDD4* mRNA and hsa-miR199a-3p. **(E)** Data from luciferase assay showed that in *NEDD4* associated assays, the luciferase activity of the *NEDD4* wild-type group was significantly inhibited by hsa-miR199a-3p. The data were presented as the mean ± SD from three independent experiments. *p < 0.05; **p < 0.01; ***p < 0.001; ns, not significant. NC, negative controls; WT, wild-type; MUT, mutant.

To identify the direct target of exosomal hsa-miR199a-3p, five miRNA target- predicting databases (miRWalk, miRanda, RNA22, Targetscan, and Tumor Suppressor Gene Database) were used. Combining the overlapped predicted targets across the five databases, 40 potential target genes of hsa-miR199a-3p were identified (listed in Table 1). Among these 40 predicted targets, *NEDD4*, a tumor suppressor gene, was selected because of its known biological functions related to NB progression. To confirm the interaction between hsa-miR199a-3p and *NEDD4*, the hsa-miR199a-3p mimic and wild-type *NEDD4* overexpression plasmid, which contains the 3′-untranslated regions (UTR) of *NEDD4*, were transfected separately or in combination into SK-N-SH cells. As determined by qRT-PCR, the expression of *NEDD4* were negatively associated with hsa-miR199a-3p (Figures 6A,B), suggesting that *NEDD4* may be taken as the acting target of the antagonist for hsa-miR199a-3p in NB. In addition, Western blotting showed the expression of *NEDD4* was down-regulated in SK-N-SH cells after transfection with hsa-miR199a-3p mimic (Figure 6C). To further demonstrate the direct regulation of *NEDD4* by hsa-miR199a-3p, the pmiR-GLO vectors with or without wild-type or mutant 3′-UTR of *NEDD4* mRNA were constructed (Figure 6D). In *NEDD4* associated assays, the luciferase activity of the *NEDD4* wild-type group was significantly inhibited by hsa-miR199a-3p (Figure 6E). These results indicate that hsa-miR199a-3p may inhibit *NEDD4* expression by binding to the 366-373 site of the 3′-UTR of *NEDD4* mRNA in NB cells.

**Table 1:** List of predicted 40 potential target genes of miR199a-3p.

**miRNA**	**MIMATid**	**Gene**	**EntrezID**	**RefseqID**	**miRWalk**	**miRanda**	**RNA22**	**Targetscan**	**SUM**
hsa-miR-199a-3p	MIMAT0000232	*KDM5A*	5927	NM_001042603	1	1	1	1	4
hsa-miR-199a-3p	MIMAT0000232	*KDM3A*	55818	NM_018433	1	1	1	1	4
hsa-miR-199a-3p	MIMAT0000232	*MAP3K4*	4216	NM_005922	1	1	1	1	4
hsa-miR-199a-3p	MIMAT0000232	*COPS2*	9318	NM_001143887	1	1	1	1	4
hsa-miR-199a-3p	MIMAT0000232	*ERBB4*	2066	XM_005246375	1	1	1	1	4
hsa-miR-199a-3p	MIMAT0000232	*ZBTB18*	10472	NM_006352	1	1	1	1	4
hsa-miR-199a-3p	MIMAT0000232	*CYLD*	1540	XM_005255811	1	1	1	1	4
hsa-miR-199a-3p	MIMAT0000232	*IL17RD*	54756	XM_005265238	1	1	1	1	4
hsa-miR-199a-3p	MIMAT0000232	*PTPRJ*	5795	NM_002843	1	1	1	1	4
hsa-miR-199a-3p	MIMAT0000232	*FAT1*	2195	XM_005262834	1	1	1	1	4
hsa-miR-199a-3p	MIMAT0000232	*PHF6*	84295	NM_032458	1	1	1	1	4
hsa-miR-199a-3p	MIMAT0000232	*PTPRC*	5788	NM_002838	1	1	1	1	4
hsa-miR-199a-3p	MIMAT0000232	*LOX*	4015	NM_002317	1	1	1	1	4
hsa-miR-199a-3p	MIMAT0000232	*CSNK1A1*	1452	XM_005268376	1	1	1	1	4
hsa-miR-199a-3p	MIMAT0000232	*ABI2*	10152	XM_005246217	1	1	1	1	4
hsa-miR-199a-3p	MIMAT0000232	*NEDD4*	4734	NM_198400	1	1	1	1	4
hsa-miR-199a-3p	MIMAT0000232	*IKZF1*	10320	NM_006060	1	1	1	1	4
hsa-miR-199a-3p	MIMAT0000232	*NF1*	4763	NM_001042492	1	1	1	1	4
hsa-miR-199a-3p	MIMAT0000232	*TRIM13*	10206	NM_213590	1	1	1	1	4
hsa-miR-199a-3p	MIMAT0000232	*LATS1*	9113	NM_004690	1	1	1	1	4
hsa-miR-199a-3p	MIMAT0000232	*RCHY1*	25898	NM_001278539	1	1	1	1	4
hsa-miR-199a-3p	MIMAT0000232	*NKX3-1*	4824	NM_006167	1	1	1	1	4
hsa-miR-199a-3p	MIMAT0000232	*DNMT3A*	1788	XM_005264175	1	1	1	1	4
hsa-miR-199a-3p	MIMAT0000232	*CTDSPL*	10217	NM_001008392	1	1	1	1	4
hsa-miR-199a-3p	MIMAT0000232	*ATM*	472	XM_005271562	1	1	1	1	4
hsa-miR-199a-3p	MIMAT0000232	*SYNPO2*	171024	NM_001128933	1	1	1	1	4
hsa-miR-199a-3p	MIMAT0000232	*KDM6A*	7403	XM_005272655	1	1	1	1	4
hsa-miR-199a-3p	MIMAT0000232	*CBL*	867	NM_005188	1	1	1	1	4
hsa-miR-199a-3p	MIMAT0000232	*DUSP5*	1847	NM_004419	1	1	1	1	4
hsa-miR-199a-3p	MIMAT0000232	*PHLPP2*	23035	XM_005255852	1	1	1	1	4
hsa-miR-199a-3p	MIMAT0000232	*PRKCB*	5579	NM_002738	1	1	1	1	4
hsa-miR-199a-3p	MIMAT0000232	*CITED2*	10370	NM_006079	1	1	1	1	4
hsa-miR-199a-3p	MIMAT0000232	*FOXA2*	3170	NM_021784	1	1	1	1	4
hsa-miR-199a-3p	MIMAT0000232	*RUNX1*	861	NM_001001890	1	1	1	1	4
hsa-miR-199a-3p	MIMAT0000232	*RARB*	5915	NM_000965	1	1	1	1	4
hsa-miR-199a-3p	MIMAT0000232	*STAT1*	6772	NM_007315	1	1	1	1	4
hsa-miR-199a-3p	MIMAT0000232	*PRKCE*	5581	XM_005264429	1	1	1	1	4
hsa-miR-199a-3p	MIMAT0000232	*RB1*	5925	NM_000321	1	1	1	1	4
hsa-miR-199a-3p	MIMAT0000232	*RECK*	8434	XM_005251613	1	1	1	1	4
hsa-miR-199a-3p	MIMAT0000232	*YAP1*	10413	XM_005271378	1	1	1	1	4

### Exosomal hsa-miR199a-3p Promotes Proliferation and Migration via *NEDD4* in NB Cells

**Figure 7:**
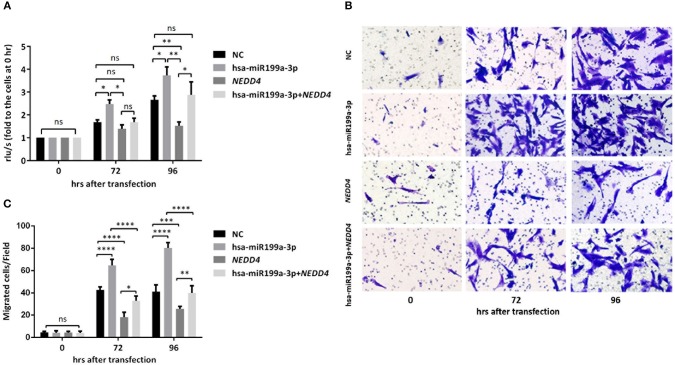
hsa-miR199a-3p promoted proliferation and migration via *NEDD4* in NB cells. **(A)** Rescue assays showed that overexpression of hsa-miR199a-3p mimic can restore the proliferation of SK-N-SH cells 96 h after transfection by antagonizing the inhibitory effects of *NEDD4*. **(B,C)** Overexpression of hsa-miR199a-3p mimic can inhibit migration capacity of SK-N-SH cells 72 and 96 h after transfection by antagonizing the inhibitory effects of *NEDD4*. Representative photographs were shown in B and quantification of the migrated cells per field was shown in **(C)**. All the data were presented as the mean ± SD from three independent experiments. *p < 0.05; **p < 0.01; ***p < 0.001; ****p < 0.0001; ns, not significant.

To further verify whether the effects of exosomal hsa-miR199a-3p on the proliferation and migration in NB are mediated by targeting *NEDD4*, rescue assays were performed. Overexpression of *NEDD4* alone in SK-N-SH cells resulted in a significant decrease in cell proliferation and migration 96 and 72 h after transfection, respectively. In contrast, simultaneous overexpression of *NEDD4* and the hsa-miR199a-3p mimic in SK-N-SH cells can restore their proliferation and migration capacity (Figure 7). Taken together, our data indicate that hsa-miR199a-3p promotes proliferation and facilitates migration by regulating *NEDD4* expression.

## Discussion

Fast, easy and non-invasive detection in NB patients is important for guiding its therapeutic strategy and assessing prognosis. So far, the biopsy of tumor tissues or bone marrows from patients suspected with NB is the golden standard for NB diagnosis (12, 13), but it is not specific enough to identify histology for the tumor heterogeneous in some cases. Although several advanced investigations that include next-generation sequencing of tumor cells and immunological assessments to define targets in the tumor microenvironment can contribute to the diagnosis and staging of NB, these detective methods are invasive, time-consuming and also difficult to repeat the procedure (14, 15). More recently, there has been a growing number of publications focusing on the function of exosomes in body fluids as an early and non-invasive diagnostic biomarker in malignant tumors. For example, serum exosomal hsa-miR-18a, hsa-miR-221, hsa-miR-222, and hsa-miR-224 have been reported as novel biomarkers for hepatocellular carcinoma (HCC) (16), and these serum exosomal miRNAs are better in distinguishing HCC from chronic hepatitis B or liver cirrhosis compared to the corresponding serum circulating miRNAs. In another study, the test of glypican-1 (GPC1) protein on cancer-cell-derived exosomes may serve as a potential diagnostic and screening tool to detect early stages of pancreatic cancer (17). Because tumor-derived exosomes have been reported to carry a cargo of molecules and factors (DNAs, miRNAs, lncRNAs and proteins) that are able to transfer information from the parent tumor cells to other cells present within and outside the tumor microenvironment (18), they are potential biomarkers for the early diagnosis of cancers. In this study, the plasma exosomal hsa-miR199a-3p was firstly reported to be upregulated in NB patients and correlated with high-risk NB, suggesting that the plasma exosomal hsa-miR199a-3p may be a suitable diagnostic and prognostic biomarker for NB in clinic without invasive operation.

Although there is no report about circulating exosomal hsa-miR199a-3p, circulating hsa-miR199a-3p has been demonstrated to play vital roles in the progression of malignant tumors. Interestingly, hsa-miR199a-3p shows opposite effects in distinct cancers (19). For instance, hsa-miR199a-3p was observed significantly lower in the tumor tissues compared with their adjacent non-tumor tissues in patients with HCC, and further research found that hsa-miR199a-3p can suppress tumor growth, migration, invasion, and angiogenesis via targeting VEGFA, VEGFR1, VEGFR2, HGF, and MMP2 proteins in HCC (20). Moreover, hsa-miR199a-3p can also restrain migration and invasion of breast cancer by downregulating PAK4/MEK/ERK signaling pathway (21). However, hsa-miR199a-3p was found to be up-regulated in gastric cancer, biliary tract cancer, and esophageal adenocarcinoma as an oncogene (22–24). In the current study, we reported that exosomal hsa-miR199a-3p can contribute to promote tumor proliferation and migration, indicating that hsa-miR199a-3p was identified as an oncogene in NB.

*NEDD4* is an HECT domain ubiquitin E3 ligase that play a critical role in the regulation of membrane receptors and endocytic machinery components (25). It is believed to be a potential dual regulator of PTEN, which exerts a tumor suppressive function by catalyzing PTEN mono-ubiquitination and regulating PTEN nuclear translocation (26). Furthermore, *NEDD4* can interact with Myc via ubiquitination and degradation of Myc protein directly. Myc protein was considered as a predictor for poor prognosis in patients with NB. Repression of *NEDD4* protein leads to the upregulation of Aurora A gene, which is important to stabilize Myc protein in NB (27). In this study, we observed that hsa-miR199a-3p caused the downregulation of *NEDD4* in NB cells and consequently promoted tumor proliferation and migration. To our best knowledge, this is the first study demonstrating that upregulation of exosomal hsa-miR199a-3p in NB can promote proliferation and migration via the inhibition of *NEDD4* expression *in vivo* and *in vitro* and lead to a poor prognosis.

There are also some limitations to the current study that need to be considered. Firstly, because of the particularity of NB, this study did not include suitable neuron-derived normal cells as controls. Second, this study is a cross-sectional study. Longitudinal studies focusing on the different periods of NB will have an important role in terms of disease progression. In addition, the sample size was limited, so larger research studies from different populations need to be conducted to confirm the findings.

In conclusion, this study reveals that exosomal hsa-miR199a-3p can promote tumor proliferation and migration via decreasing *NEDD4* expression in NB patients and cells. Our study elucidates a new molecular mechanism concerning the initiation and progression of NB, which indicates that exosomal hsa-miR199a-3p can be applicated as a fast, easy and non-invasive detection biomarker and also can contribute to investigate novel therapeutic strategies for NB.

## Ethics Statement

This study was carried out in accordance with the 1964 Declaration of Helsinki and its later amendments with written informed consent from all subjects. The protocol was approved by the Institutional Review Board and the Ethics Committee of SCMC (SCMCIRB-K2017042), and written informed consent was obtained from the parents of each participant.

## Author Contributions

XM, SG, and NS conceived the project, analyzed the data and wrote the manuscript. JM, MX, MY, JH, HC, YG, and CX performed the experiments and analyzed the data.

### Conflict of Interest Statement

The authors declare that the research was conducted in the absence of any commercial or financial relationships that could be construed as a potential conflict of interest.

## References

[1] Decarolis B Simon T Krug B Leuschner I Vokuhl C Kaatsch P. Treatment and outcome of Ganglioneuroma and Ganglioneuroblastoma intermixed. BMC Cancer. (2016) 16:542. 10.1186/s12885-016-2513-9 27465021 PMC4964292

[2] Mora J Gerald WL. Origin of neuroblastic tumors: clues for future therapeutics. Expert Rev Mol Diagn. (2004) 4:293–302. 10.1586/14737159.4.3.293 15137897

[3] Ward E DeSantis C Robbins A Kohler B Jemal A. Childhood and adolescent cancer statistics, 2014. CA Cancer J Clin. (2014) 64:83–103. 10.3322/caac.21219 24488779

[4] Louis CU Shohet JM. Neuroblastoma: molecular pathogenesis and therapy. Annu Rev Med. (2015) 66:49–63. 10.1146/annurev-med-011514-023121 25386934 PMC4418018

[5] Ahmed AA Zhang L Reddivalla N Hetherington M. Neuroblastoma in children: update on clinicopathologic and genetic prognostic factors. Pediatr Hematol Oncol. (2017) 34:165–85. 10.1080/08880018.2017.1330375 28662353

[6] Hannafon BN Trigoso YD Calloway CL Zhao YD Lum DH Welm AL. Plasma exosome microRNAs are indicative of breast cancer. Breast Cancer Res. (2016) 18:90. 10.1186/s13058-016-0753-x 27608715 PMC5016889

[7] Felicetti F De Feo A Coscia C Puglisi R Pedini F Pasquini L. Exosome-mediated transfer of miR-222 is sufficient to increase tumor malignancy in melanoma. J Transl Med. (2016) 14:56. 10.1186/s12967-016-0811-2 26912358 PMC4765208

[8] Vaksman O Trope C Davidson B Reich R. Exosome-derived miRNAs and ovarian carcinoma progression. Carcinogenesis. (2014) 35:2113–20. 10.1093/carcin/bgu130 24925027

[9] Haug BH Hald OH Utnes P Roth SA Lokke C Flaegstad T. Exosome-like extracellular vesicles from MYCN-amplified neuroblastoma cells contain oncogenic miRNAs. Anticancer Res. (2015) 35:2521–30. 25964525

[10] Challagundla KB Wise PM Neviani P Chava H Murtadha M Xu T. Exosome-mediated transfer of microRNAs within the tumor microenvironment and neuroblastoma resistance to chemotherapy. J Natl Cancer Inst. (2015) 107:djv135. 10.1093/jnci/djv135 25972604 PMC4651042

[11] Thery C Amigorena S Raposo G Clayton A. Isolation and characterization of exosomes from cell culture supernatants and biological fluids. Curr Protoc Cell Biol. (2006) Chapter 3:Unit 3 22. 10.1002/0471143030.cb0322s30 18228490

[12] Burchill SA Beiske K Shimada H Ambros PF Seeger R Tytgat GA. Recommendations for the standardization of bone marrow disease assessment and reporting in children with neuroblastoma on behalf of the International Neuroblastoma Response Criteria Bone Marrow Working Group. Cancer. (2017) 123:1095–105. 10.1002/cncr.30380 27984660

[13] Park JR Bagatell R Cohn SL Pearson AD Villablanca JG Berthold F. Revisions to the international neuroblastoma response criteria: a consensus statement from the national cancer institute clinical trials planning meeting. J Clin Oncol. (2017) 35:2580–7. 10.1200/JCO.2016.72.0177 28471719 PMC5676955

[14] Pugh TJ Morozova O Attiyeh EF Asgharzadeh S Wei JS Auclair D. The genetic landscape of high-risk neuroblastoma. Nat Genet. (2013) 45:279–84. 10.1038/ng.2529 23334666 PMC3682833

[15] Asgharzadeh S Salo JA Ji L Oberthuer A Fischer M Berthold F. Clinical significance of tumor-associated inflammatory cells in metastatic neuroblastoma. J Clin Oncol. (2012) 30:3525–32. 10.1200/JCO.2011.40.9169 22927533 PMC3675667

[16] Sohn W Kim J Kang SH Yang SR Cho JY Cho HC. Serum exosomal microRNAs as novel biomarkers for hepatocellular carcinoma. Exp Mol Med. (2015) 47:e184. 10.1038/emm.2015.68 26380927 PMC4650928

[17] Melo SA Luecke LB Kahlert C Fernandez AF Gammon ST Kaye J. Glypican-1 identifies cancer exosomes and detects early pancreatic cancer. Nature. (2015) 523:177–82. 10.1038/nature14581 26106858 PMC4825698

[18] Al-Nedawi K Meehan B Micallef J Lhotak V May L Guha A. Intercellular transfer of the oncogenic receptor EGFRvIII by microvesicles derived from tumour cells. Nat Cell Biol. (2008) 10:619–24. 10.1038/ncb1725 18425114

[19] Gu S Chan WY. Flexible and versatile as a chameleon-sophisticated functions of microRNA-199a. Int J Mol Sci. (2012) 13:8449–66. 10.3390/ijms13078449 22942713 PMC3430244

[20] Ghosh A Dasgupta D Ghosh A Roychoudhury S Kumar D Gorain M. MiRNA199a-3p suppresses tumor growth, migration, invasion and angiogenesis in hepatocellular carcinoma by targeting VEGFA, VEGFR1, VEGFR2, HGF and MMP2. Cell Death Dis. (2017) 8:e2706. 10.1038/cddis.2017.123 28358369 PMC5386529

[21] Li SQ Wang ZH Mi XG Liu L Tan Y. MiR-199a/b-3p suppresses migration and invasion of breast cancer cells by downregulating PAK4/MEK/ERK signaling pathway. IUBMB Life. (2015) 67:768–77. 10.1002/iub.1433 26399456

[22] Ueda T Volinia S Okumura H Shimizu M Taccioli C Rossi S. Relation between microRNA expression and progression and prognosis of gastric cancer: a microRNA expression analysis. Lancet Oncol. (2010) 11:136–46. 10.1016/S1470-2045(09)70343-2 20022810 PMC4299826

[23] Shigehara K Yokomuro S Ishibashi O Mizuguchi Y Arima Y Kawahigashi Y. Real-time PCR-based analysis of the human bile microRNAome identifies miR-9 as a potential diagnostic biomarker for biliary tract cancer. PLoS ONE. (2011) 6:e23584. 10.1371/journal.pone.0023584 21858175 PMC3157401

[24] Feber A Xi L Pennathur A Gooding WE Bandla S Wu M. MicroRNA prognostic signature for nodal metastases and survival in esophageal adenocarcinoma. Ann Thorac Surg. (2011) 91:1523–30. 10.1016/j.athoracsur.2011.01.056 21420070 PMC3399250

[25] Boase NA Kumar S. *NEDD4*: the founding member of a family of ubiquitin-protein ligases. Gene. (2015) 557:113–22. 10.1016/j.gene.2014.12.020 25527121 PMC6639052

[26] Trotman LC Wang X Alimonti A Chen Z Teruya-Feldstein J Yang H. Ubiquitination regulates PTEN nuclear import and tumor suppression. Cell. (2007) 128:141–56. 10.1016/j.cell.2006.11.040 17218261 PMC1855245

[27] Sun Y Liu PY Scarlett CJ Malyukova A Liu B Marshall GM. Histone deacetylase 5 blocks neuroblastoma cell differentiation by interacting with N-Myc. Oncogene. (2014) 33:2987–94. 10.1038/onc.2013.253 23812427

