# Supplementary material for: Exosomal hsa-miR199a-3p Promotes Proliferation and Migration in Neuroblastoma
Source: Front Oncol. 2019 Jun 12;9:459. doi: 10.3389/fonc.2019.00459 (PMC6582313; doi:10.3389/fonc.2019.00459)
Supplement: Supplementary file 1 [file Data_Sheet_1.docx]

***Supplementary Materials***

**Supplementary Tables**

**Table S1.** Clinical-pathologic characteristics of the 15 patients with NB and GNBi for sequencing cohort

| **Characteristics** | **Number of cases（%）** |
| --- | --- |
| **Age(m)** |  |
| > 18 | 11 (73.3) |
| < 18 | 4 (26.7) |
| **Gender** |  |
| Male | 6 (40) |
| Female | 9 (60) |
| **INSS** |  |
| I | 2 (13.3) |
| II | 4 (26.7) |
| III | 5 (33.3) |
| IV | 4 (26.7) |
| IVs | 0 (0) |
| **INPC** |  |
| FH | 12 (80) |
| UFH | 3 (20) |
| **COG risk criteria** |  |
| Low | 4 (26.7) |
| Intermediate | 6 (40) |
| High | 5 (33.3) |
| **Lymphatic invasion** |  |
| Yes | 7 (46.7) |
| No | 8 (53.3) |

Abbreviations: INSS, International Neuroblastoma Staging System; INPC, International Neuroblastoma Pathology Classification; FH, favorable histology; UFH, unfavorable histology; COG, Children's Oncology Group.

**Table S2.** Clinical-pathologic characteristics of the 8 patients with NB for validation cohort

| **Characteristics** | **Number of cases（%）** |
| --- | --- |
| **Age(m)** |  |
| > 18 | 6 (75) |
| < 18 | 2 (25) |
| **Gender** |  |
| Male | 3 (37.5) |
| Female | 5 (62.5) |
| **INSS** |  |
| I | 1 (12.5) |
| II | 3 (37.5) |
| III | 2 (25) |
| IV | 2 (25) |
| IVs | 0 (0) |
| **INPC** |  |
| FH | 6 (75) |
| UFH | 2 (25) |
| **COG risk criteria** |  |
| Low | 3 (37.5) |
| Intermediate | 2 (25) |
| High | 3 (37.5) |
| **Lymphatic invasion** |  |
| Yes | 4 (50) |
| No | 4 (50) |

Abbreviations: INNS, International Neuroblastoma Staging System; INPC, International Neuroblastoma Pathology Classification ; FH, favorable histology ; UFH, unfavorable histology; COG, Children's Oncology Group.

**Table S3.** Clinical-pathologic characteristics of the 18 patients with NB and GNBi for fresh tumor tissue

| **Characteristics** | **Number of cases（%）** |
| --- | --- |
| **Age(m)** |  |
| > 18 | 12 (66.7) |
| < 18 | 6 (33.3) |
| **Gender** |  |
| Male | 7 (38.9) |
| Female | 11 (61.1) |
| **INSS** |  |
| I | 3 (16.7) |
| II | 8 (44.4) |
| III | 4 (22.2) |
| IV | 3 (16.7) |
| IVs | 0 (0) |
| **INPC** |  |
| FH | 14 (77.8) |
| UFH | 4 (22. 2) |
| **COG risk criteria** |  |
| Low | 4 (22.2) |
| Intermediate | 8 (44.4) |
| High | 6 (33.3) |
| **Lymphatic invasion** |  |
| Yes | 7 (38.9) |
| No | 11 (61.1) |

Abbreviations: INNS, International Neuroblastoma Staging System; INPC, International Neuroblastoma Pathology Classification ; FH, favorable histology ; UFH, unfavorable histology; COG, Children's Oncology Group.
